# Susceptibility of *Escherichia coli* O157:H7 grown at low temperatures to the krypton-chlorine excilamp

**DOI:** 10.1038/s41598-018-37060-1

**Published:** 2019-01-24

**Authors:** Jae-Ik Lee, Sang-Soon Kim, Dong-Hyun Kang

**Affiliations:** 1 Department of Agricultural Biotechnology, Center for Food and Bioconvergence, and Research Institute for Agricultural and Life Sciences, Seoul National University, Seoul, 08826 Republic of Korea; 2 Institutes of Green Bio Science & Technology, Seoul National University, Pyeongchang-gun, Gangwon-do 25354 Republic of Korea

## Abstract

This study was conducted to investigate the resistance of *Escherichia coli* O157:H7 to 222-nm krypton-chlorine(KrCl) excilamp and 254-nm low-pressure Hg lamp (LP lamp) treatment according to growth temperature. As growth temperature decreased, lag time of *E. coli* O157:H7 significantly increased while the growth rate significantly decreased. Regardless of growth temperature, the KrCl excilamp showed higher disinfection capacity compared to the LP lamp at stationary growth phase. KrCl excilamp treatment showed significantly higher reduction as growth temperature decreased. Conversely, reduction levels according to growth temperature were not significantly different when the pathogen was subjected to LP lamp treatment. Inactivation mechanisms were evaluated by the thiobarbituric acid reactive substances (TBARS) assay and SYBR green assay, and we confirmed that lipid oxdiation capacity following KrCl excilamp treatment increased as growth temperature decreased, which was significantly higher than that of LP lamp treated samples regardless of growth temperature. DNA damage level was significantly higher for LP Hg lamp treated samples compared to those subjected to the KrCl excilamp, but no significant difference pursuant to growth temperature was observed. At the transcriptional level, gene expression related to several metabolic pathways was significantly higher for the pathogen grown at 15 °C compared that of 37 °C, enabling it to adapt and survive at low temperature, and membrane lipid composition became altered to ensure membrane fluidity. Consequently, resistance of *E. coli* O157:H7 to the KrCl excilamp decreased as growth temperature decreased because the ratio of unsaturated fatty acid composition increased at low growth temperature resulting in higher lipid oxidation levels. These results indicate that KrCl excilamp treatment should be determined carefully considering the growth temperature of *E. coli* O157:H7.

## Introduction

*Escherichia coli* O157:H7 has been associated with 350 outbreaks over a twenty year period^1^. This pathogen has caused severe public health and economic problems worldwide with numerous high profile outbreaks (CDC). One of the biggest risks of *E. coli* O157:H7 is related to its low infective doses^2^. Ingestion of as few as 10 organisms may be sufficient to cause infection, which results in bloody diarrhea and hemolytic uremic syndrome^3^. Because cross-contamination during processing commonly occurs from utensils and surfaces^4^, even slight contamination of the pathogens onto surfaces or work areas may cause serious outbreaks^5^. For example, stainless steel, which is still widely used for constructing processing equipment, can be contaminated with the pathogen. Several research investigations reported that *E. coli* O157:H7 can survive on stainless steel for an extended time (28–60 days) at low temperatures^6,7^. These results indicate that the pathogen can survive on stainless steel at numerous temperatures and result in cross contamination from stainless steel to food or medical equipment. Through the present time, sodium hypochlorite (NaOCl) has been used widely to prevent outbreaks and sanitize stainless steel in numerous facilities. However, several disadvantages of NaOCl have been reported including, corrosion of a variety of substances^8^.

In recent years, several research studies have reported on the inactivation of *E. coli* O157:H7 on stainless steel using ultraviolet-C as an alternative to NaOCl^4,9,10^. Ultraviolet-C is one of the non-thermal technologies approved by the U.S. Food and Drug Administration, and widely used to sterilize food surfaces, equipment as well as water. Ultraviolet-C ranges 200 to 280 nm, and it is widely known that 254 nm is the most effective wavelength for disinfecting bacteria within this range. When DNA absorbs UV light at 254 nm, nucleotide base pairing is altered, thereby creating new linkages on DNA strands. As a result, DNA replication is blocked which eventually leads to cell death^11^. Therefore, low pressure mercury lamps (LP lamp) have been used widely to provide monochromatic 254 nm irradiation for the food and clinical industry. Even though LP lamps are cheap and energy efficient, they have critical drawbacks. Their disadvantages include a long warm-up time, a short life time and risk of mercury leakage. In particular, mercury leakage from LP lamps can cause severe human health hazards as well as environmental damage. Moreover, a new international binding treaty instrument called the Minamata Convention was approved, which restricts the usage of mercury^12^. For the reasons given above, the necessity of developing a new source of ultraviolet-C irradiation has been emphasized recently. In the last decades, dielectric barrier discharge (DBD)-driven excilamps (excimer or exciplex lamp) have been introduced as an alternative to LP lamps. Excilamps utilize the non-equilibrium radiation of excimer and exciplex molecules^13^, and emit a narrow band wavelength range from 172 to 345 nm^14^. The merits of excilamps compared to LP lamps include absence of mercury, longer life time, wavelength-selective application and so on^15^. Recently, numerous attempts have been made by researchers to show bacterial disinfection by excilamps. G. Matafonova *et al*.^14^ reported that the KrCl excilamp (222 nm) effectively inactivates gram positive and gram negative bacteria in liquid suspensions. Also, inactivation of *E. coli* O157:H7 in apple juice at 222 nm was higher than at 282 nm and at 254 nm at similar levels of UV fluence^16^. Besides in liquid samples, Ha *et al*.^17^ indicated that the KrCl excilamp is more effective than the LP lamp on solid surfaces. In practice, *E. coli* O157:H7 encounters various environments such as high/low temperatures, diverse pH, and antibiotics. To survive and grow, *E. coli* O157:H7 has adapted to various environments via responses that include in DNA supercoiling, regulating production of protein, fatty acid composition, and DNA transcription^18,19^. Within various environmental parameters, temperature has been recognized as a cardinal factor controlling microbial population^20^. A great deal of inquiry has been directed toward high/low temperature adaptation of *E. coli* O157:H7^21,22^. However, a large number of studies have been made at optimum or higher temperature^23,24^ and focused on the virulence factor, stress response, and gene expression^25–27^.

The purpose of this study was to identify the resistance of *E. coli* O157:H7 grown at various temperatures to LP lamp and KrCl excilamp treatment. Lipid oxidation, and DNA damage to *E. coli* O157:H7 were compared for each growth temperatures to identify the inactivation mechanisms. Moreover, transcriptome and lipid composition of *E. coli* O157:H7 were analyzed in the present study.

## Results

### Growth curves of *E. coli* O157:H7 after growing at different temperatures

**Figure 1:**
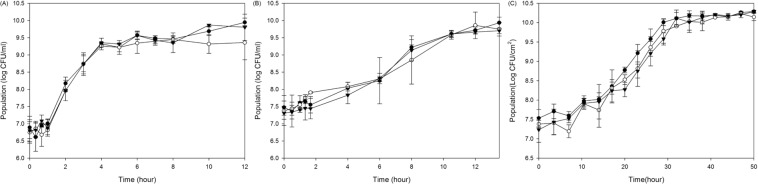
Growth curves of *E. coli* O157:H7 35150 (●), ATCC 43889 (○), and ATCC 43890 (▼) grown at 37 °C (**A**), 25 °C (**B**), and 15 °C (**C**).

Temperature has a significant effect on the growth of *E. coli* O157:H7 (Fig. 1). Lag time and growth rate were significantly affected by temperature while final cell numbers were not significantly different. Lag time increased as temperature decreased. The mean lag times were 0.9, 2.7, and 11.5 h for *E. coli* O157:H7 grown at 37, 25, and 15 °C, respectively. On the other hand, growth rate decreased as temperature decreased. The incubation times for the pathogens to enter stationary phase were 6, 12, and 42 h for 37, 25, and 15 °C, respectively. It is widely accepted that stationary phase is the stage most resistant to at various stresses. Jenkins *et al*.^28^ found that stationary phase cells are extremely resistant to various stresses and inimical processes because the *rpoS* gene is expressed at stationary phase, which is associated with many stress resistance gene such as *appR* (acid phosphatase expression), *nur* (near UV resistance) and *csi*(carbon starvation)^29^. Therefore, we investigated the resistance of *E. coli* O157:H7 to the LP lamp or KrCl excilamp at stationary phase. Final cell numbers at the stationary phase were about 10^10,11^ CFU/ml regardless of growth temperature.

### Reduction of *E. coli* O157:H7

Growth temperature had a significant effect on the inactivation of *E. coli* O157:H7 by KrCl excilamp treatment while significant differences were not observed in LP lamp treated cells (Table 1). Reduction levels (log CFU/cm^2^) of *E. coli* O157:H7 (mixed culture of ATCC 35150, ATCC 43889, and ATCC 43890) subjected to KrCl excilamp exposure increased as growth temperature decreased at same dose treatment (mJ/cm^2^). When *E. coli* O157:H7, grown at 37, 25, and 15 °C were, subjected to the KrCl excilamp at an dose of 156.6 mJ/cm^2^, the reduction levels (log CFU/cm^2^) enumerated on SMAC were 2.48, 3.12, and 4.74, respectively. This same tendency was observed when *E. coli* O157:H7 was enumerated on SPRAB, and significant generation of injured cells was not observed (<1 log) for all treatment conditions. At the identical dose, KrCl excilamp treatment more effectively inactivated *E. coli* O157:H7 than did LP lamp treatment.

**Table 1:** Reduction (log CFU/cm^2^) of *E. coli* O157:H7 (mixed culture of ATCC 35150, ATCC 43889, and ATCC 43890) subjected to LP lamp and KrCl excilamp treatment after growth at 37 °C, 25 °C, and 15 °C^a,b^.

Dose (mJ/cm^2^)	Treatments	SMAC			SPRAB		
		37 °C	25 °C	15 °C	37 °C	25 °C	15 °C
26.1	LP lamp	0.25 ± 0.08 Aa	0.23 ± 0.21 Aa	0.11 ± 0.20 Aa	0.21 ± 0.03 Aa	0.24 ± 0.12 Aa	0.26 ± 0.20 Aa
	KrCl excilamp	0.79 ± 0.15 Ba	1.01 ± 0.27 Ba	1.19 ± 0.50 Ba	0.81 ± 0.21 Ba	1.10 ± 0.44 Ba	1.15 ± 0.18 Ba
69.6	LP lamp	0.53 ± 0.28 Aa	0.29 ± 0.25 Aa	0.23 ± 0.28 Aa	0.26 ± 0.05 Aa	0.63 ± 0.23 Aa	0.56 ± 0.28 Aa
	KrCl excilamp	1.46 ± 0.43 Ba	1.44 ± 0.48 Ba	1.83 ± 0.38 Ba	1.25 ± 0.49 Ba	1.85 ± 0.83 Ba	1.74 ± 0.20 Ba
131.3	LP lamp	0.65 ± 0.30 Aa	0.78 ± 0.24 Aa	0.76 ± 0.55 Aa	0.35 ± 0.06 Aa	0.97 ± 0.14 Aa	0.88 ± 0.55 Aa
	KrCl excilamp	1.72 ± 0.38 Ba	2.47 ± 0.11 Bb	2.79 ± 0.20 Bb	1.69 ± 0.19 Ba	2.32 ± 1.09 Ba	2.73 ± 0.46 Ba
156.6	LP lamp	0.74 ± 0.21 Aa	1.15 ± 0.22 Aa	0.94 ± 0.47 Aa	0.42 ± 0.07 Aa	1.31 ± 0.06 Ab	1.14 ± 0.47 Ab
	KrCl excilamp	2.48 ± 0.26 Ba	3.12 ± 0.21 Bb	4.74 ± 0.33 Bc	2.17 ± 0.12 Ba	2.80 ± 0.64 Ba	4.19 ± 0.32 Bb

^a^Values in the same column for each dose followed by the same uppercase letter are not significantly different (*p* > 0.05).

^b^Values in the same row for each enumeration medium followed by the same lowercase letter are not significantly different (*p* > 0.05).

### Lipid oxidation and DNA damage

Growth temperature had a significant effect (*p* < 0.05) on lipid oxidation and DNA damage values (arbitary fluorescence units, AFU) of KrCl excilamp-treated *E. coli* O157:H7 (Tables 2 and 3). The lipid oxidation values were 10.74, 14.26, and 28.97 AFU for 37 °C, 25 °C and 15 °C grown cells, respectively. The DNA damage values were 1.06, 1.15 and 4.64 AFU for 37 °C, 25 °C and 15 °C grown cells, respectively. On the other hand, growth temperature had no significant effect (*p* > 0.05) on either lipid oxidation or DNA damage of LP lamp-treated *E. coli* O157:H7. Lipid oxidation values were significantly higher (*p* < 0.05) for KrCl excilamp- treated cells than for LP lamp-treated cells while DNA damage values were significantly higher (*p* < 0.05) for LP lamp-treated cells than for KrCl excilamp-treated cells.

**Table 2:** Lipid oxidation values (arbitary fluorescence units, AFU) of *E. coli* O157:H7 ATCC 35150 subjected to KrCl excilamp and LP lamp treatment after growth at 37 °C, 25 °C and 15 °C^a,b^.

	37 °C	25 °C	15 °C
LP lamp	0.20 ± 0.59 Aa	0.38 ± 0.19 Aa	0.35 ± 0.23 Aa
KrCl excilamp	10.74 ± 2.00 Ba	14.26 ± 0.54 Bb	28.97 ± 2.11 Bc

Mean values ± standard deviation.

^a^Values in the same column followed by the same uppercase letter are not significantly different (*p* > 0.05).

^b^Values in the same row followed by the same lowercase letter are not significantly different (*p* > 0.05).

**Table 3:** DNA damage values (arbitary fluorescence units, AFU) of *E. coli* O157:H7 ATCC 35150 subjected to KrCl excilamp and LP lamp treatment after grown at 37 °C, 25 °C and 15 °C^a,b^.

	37 °C	25 °C	15 °C
LP lamp	6.61 ± 1.64 Aa	8.59 ± 1.34 Aa	8.41 ± 2.03 Aa
KrCl excilamp	1.06 ± 0.82 Ba	1.15 ± 0.68 Ba	4.64 ± 0.89 Bb

Mean values ± standard deviation.

^a^Values in the same column followed by the same uppercase letter are not significantly different (*p* > 0.05).

^b^Values in the same row followed by the same lowercase letter are not significantly different (*p* > 0.05).

### Transcriptome analysis of *E. coli* O157:H7 35150

**Figure 2:**
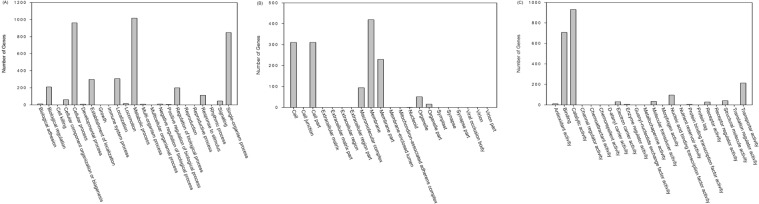
Number of differentially expressed genes of *E. coli* O157:H7 35150 for biological process (**A**), cellular component (**B**), and molecular function (**C**) in GO category.

**Figure 3:**
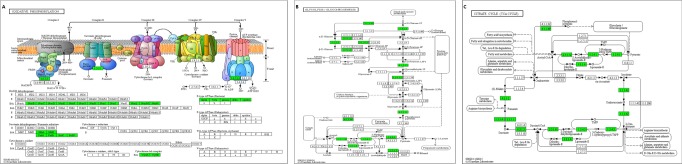
Down-regulated genes (green) in the pathway of oxidative phosphorylation (**A**), glycolysis/glucogenesis (**B**), and citrate cycle (**C**). Pathway analysis of the selected genes was performed using the KEGG database (http://www.genome.ad.jp/kegg)^46–48^.

**Figure 4:**
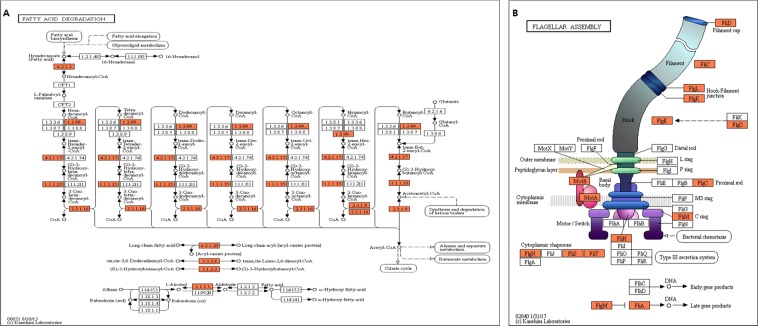
Up-regulated genes (orange) in the pathway of fatty acid degradation (**A**) and flagella assembly (**B**). Pathway analysis of the selected genes was performed using the KEGG database (http://www.genome.ad.jp/kegg)^46–48^.

Transcriptome of *E. coli* O157:H7 ATCC 35150 grown at 15 °C was compared with that grown at 37 °C. Differentially expressed genes (DEGs) were annotated in the GO database, which is a standardized system for gene functional classfication, containing 3 domains that are classified by biological process, cellular components, and molecular functions (Fig. 2). In the category of biological processes, most DEGs were related to the cellular process, metabolic process, and single-organism processes. In the category of cellular components, most DEGs were related to the cell (including cell part) and membrane (including membrane part). Finally in the category of molecular functions, most DEGs were related to binding and catalytic activity. We selected several significant pathways by analyzing up-regulated and down-regulated genes. A variety of genes related to oxidative phosphorylation (Fig. 3A), glycolysis/glucogenesis (Fig. 3B), and citrate cycle (Fig. 3C) were down-regulated. On the other hand, a variety of genes related to fatty acid degradation (Fig. 4A) and flagella assembly (Fig. 4B) were up-regulated.

### Membrane fatty acid composition

**Figure 5:**
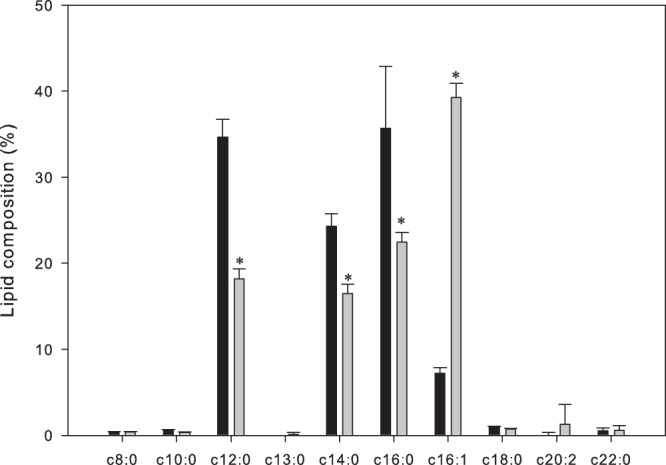
Membrane fatty acid composition of *E. coli* O157:H7 ATCC 35150 after grown at 37 °C (■) and 15 °C (■). The data represent the mean ± standard deviation (SD, n = 3).

The ratio of unsaturated fatty acids (USFA) to saturated fatty acids (SFA) was higher for the *E. coli* O157:H7 ATCC 35150 membrane grown at lower rather than higher temperatures (Fig. 5). Relative contents (%) of palmitoleic acid of the cell membrane was 7.2 and 39.3 for *E. coli* O157:H7 ATCC 35150 grown at 37 °C and 15 °C, respectively. On the other hand, relative content of lauric, myristic, and palmitic acids decreased as growth temperature decreased. The decrements (%) were 16.4, 7.8, and 13.2 for lauric, myristic, and palmitic acid, respectively, when growth temperature decreased from 37 °C to 15 °C. Growth temperature of *E. coli* O157:H7 ATCC 35150 had no significant effect (*p* > 0.05) on the relative content of the rest of the fatty acids.

## Discussion

The KrCl excilamp, a new source of UV-C emitter, has been demonstrated to be effective for controlling major foodborne pathogens on solid surfaces^17^. In the present study, we identified that resistance of *E. coli* O157:H7 to high dose (>131.3 mJ/cm^2^) KrCl excilamp treatment was significantly different depending on growth temperature. It seems that sufficient dose (>hreshold value) is needed to induce the significant difference. Reduction levels (log CFU/cm^2^) of *E. coli* O157:H7 on stainless steel subjected to high dose KrCl excilamp was higher for cells grown at lower temperature (Table 1). We assumed that specific bactericidal targets of *E. coli* O157:H7, such as DNA, proteins, or lipids are affected by growth temperature. At first, we investigated protein photodegradation, which has been revealed as a one of the bactericidal mechanisms of the KrCl excilamp^17,30^. However, significant differences were not observed when we quantified protein concentrations of *E. coli* O157:H7 after growing it at various temperatures (data not shown). Therefore, protein degradation is not sufficient to describe different reduction levels of *E. coli* O157:H7 grown at different temperatures. Recently, it was found that KrCl excilamp treatment can produce reactive oxygen species resulting in lipid oxidation^31^. Gomez *et al*.^32^ found that KrCl excilamp can utilize advanced oxidation processes. Also, lipid oxidation was observed in KrCl excilamp-treated samples, which was significantly higher than that of LP lamp-treated samples. Moreover, lipid oxidation levels of *E. coli* O157:H7 subjected to KrCl excilamp treatment increased as growth temperature decreased. Simultaneously, DNA damage levels were not significantly different relative to growth temperature. From the results above, we postulate that differing lipid oxidation levels is the major reason for different reduction levels of *E. coli* O157:H7 grown at different temperatures when subjected to KrCl excilamp treatment. In contrast to the KrCl excilamp, reduction of *E. coli* O157:H7 grown at different temperatures was not significantly different when subjected to the LP lamp. Because the results can be attributed to low reduction levels (<1.5 log), we confirmed that significant differences were not observed at high reduction levels (about 4 log) relative to the growth temperature (data not shown). The major bactericidal mechanism of LP lamps is known to be DNA damage^11^, and we identified that DNA damage values were significantly larger for LP lamp treated samples compared to KrCl excilamp treated samples regardless of growth temperature. Because the amount of DNA did not vary according to growth temperature, damage to DNA of *E. coli* O157:H7 subjected to the LP lamp did not differ significantly according to growth temperatures. This is why reduction levels by the LP lamp were not significantly different (*p* > 0.05) relative to growth temperature.

We assumed that transcriptional differences between *E. coli* O157:H7 grown at different temperatures affected the degree of lipid oxidation during KrCl excilamp exposure. Therefore, transcriptome of *E. coli* O157:H7 ATCC 35150 grown at 15 °C was compared with that grown at 37 °C. A number of DEGs were observed for *E. coli* O157:H7 grown at 15 °C compared to that grown at 37 °C. Among biological processes, metabolic and cellular processes have the largest number of DEGs. We could identify the metabolic process of *E. coli* O157:H7 at stationary phase, by using the KEGG pathway, which represents a network of interacting molecules. In the present study, TSB was used as the bacterial culture medium, which is rich in glucose as the sole carbon source. Generally, *E. coli* utilizes glucose during exponential growth phase and then acetate, which is produced as a major product during aerobic fermentation, and is used as a carbon source when glucose becomes depleted. When acetate is also used up, amino acids are utilized by *E. coli* O157:H7 during stationary phase^33^ In the present study, genes related to glucose metabolic pathway such as glycolysis, TCA cycle and oxidative phosphorylation are down-regulated in *E. coli* O157:H7 grown at 15 °C compared to 37 °C. On the other hand, genes related to fatty acid degradation and the fatty acid metabolic pathway is up-regulated for this pathogen grown at low temperature. Moreover, TesA and TesB, which catalyze long chain fatty acyl CoA such as palmitoyl-CoA, stearoyl-CoA and Oleoyl-CoA to long chain fatty acids, are upregulated by a 2.09 and 1.91 log2 fold change, respectively (Table S1). Additionally, *fadL* plays a central role for the uptake of exogenous long chain fatty acid at the outer membrane, and *fadD* encodes an inner membrane long chain fatty acid CoA ligase^34^, up-regulated to a 3.8 and 4.74 log2 fold change, respectively (Table S1). These results indicate that *E. coli* O157:H7 grown at 15 °C to stationary phase obtains energy by utilizing fatty acids as a carbon source. Because fatty acids are in a highly reduced state and less oxygenated than other carbon sources, a high amount of metabolic energy can be produced by fatty acid metabolism. Farewell *et al*.^35^ also found that fatty acid metabolism is an important part of the adaptation to stationary phase. From the results above, we submit that *E. coli* O157:H7 growing at low temperature needs fatty acid metabolism to survive in an environment of low protein concentration and low reaction rate with substrates compared to the optimal growth temperature.

Cell motility and membrane fluidity of *E. coli* O157:H7 is also known to be important for adapting to a low temperature environment. We observed that *E. coli* O157:H7 increases flagellar motility (Fig. 4B) and altered membrane fatty acid composition when grown at low temperature (Fig. 5). Because membrane fatty acid composition changes during lag phase at low temperatures^36^, transcriptional differences related to fatty acyl synthesis was not observed at stationary phase in the present study. However, we identified that palmitoleic acid content was significantly larger for *E. coli* O157:H7 grown at 15 °C than that grown at 37 °C. On the other hand, saturated fatty acid, lauric acid, myristic acid, and palmitic acid content was significantly reduced for *E. coli*O157:H7 grown at 15 °C compared to that grown at 37 °C. Because cis-unsaturated bonds make a “kink” into the acyl chain, contributing to molecular packing and molecular motions^37^. membrane fluidity increases as the ratio of unsaturated to saturated fatty acid increases. Regrading motility and fluidity, as temperature decreased, *E. coli* O157:H7 coped with active flagellar movement and an increased proportion of unsaturated fatty acyl. However, lipid oxidation reacts more easily as the degree of *cis*-unsaturated fatty acid increases. Therefore, membrane lipids of *E. coli* O157:H7 grown at low temperatures oxidized more easily than those grown at 37 °C, and consequently were more susceptible to KrCl excilamp treatment.

In conclusion, gene expression of *E. coli* O157:H7 changed to adapt and survive at low growth temperatures. As a result, membrane lipid composition was altered to ensure membrane fluidity. Because unsaturated fatty acid composition of *E. coli* O157:H7 grown at low temperature increased, lipid oxidation level increased when *E. coli* O157:H7 was subjected to KrCl excilamp treatment. Consequently, resistance of *E. coli* O157:H7 to the KrCl excilamp decreased as growth temperature decreased while the resistance was not significantly different for the LP lamp. Because *E. coli* O157:H7 is a major foodborne pathogen which causes severe risk to public health and to the economy, adequate treatment is necessary to control this pathogen. The KrCl excilamp is a promising non-thermal treatment to replace conventional LP lamps. However, excessive treatment causes quality degradation of treated samples as well as leads to economic loss. Therefore, adequate treatment time for the KrCl excilamp should be determined considering the growth temperature of *E. coli* O157:H7.

## Material and Methods

### Growth conditions

Three strains each of *E. coli* O157:H7 (ATCC 35150, ATCC 43889, ATCC 43890) were obtained from the bacteria culture collection of Seoul National University (Seoul, South Korea). A single colony cultivated from frozen stocks on tryptic soy agar (TSA; Difco, Becton, Dickinson, Sparks, MD) was inoculated into 5 ml of tryptic soy broth (TSB; Difco, Becton, Dickinson, Sparks, MD), incubated for overnight in a shaking incubator at 37 °C and 250 rpm. Optical density at 600 nm of the cell suspension was determined and adjusted to 0.1 (ca. 10^6^ CFU/ml) with 50 ml TSB. Approximately equal populations of cell suspension were combined with TSB, and incubated in a shaking incubator at different temperature (15, 25, and 37 °C) and 250 rpm.

### Growth curves

Growth of *E. coli* O157:H7 was determined by withdrawing 200 μl of samples at appropriate time intervals. Absorbance of samples was determined with a spectrofluorophotometer (Spectramax M2e; Molecular Devices, Sunnyvale, CA) at 600 nm (data not shown). Populations of viable cells withdrawn at the adjusted time intervals (based on absorbance data) were enumerated by plating on Sorbitol MacConkey (SMAC) agar (Difco). Plate counts were used to determine early stationary phase, which was 52, 12, and 7 h for 15, 25, and 37 °C, respectively.

### Cell suspension and inoculation

Cell suspensions, incubated at different temperatures, were collected by centrifugation at 4,000 × g for 20 min at 4 °C. The pellets were resuspended in 0.2% PW. The concentration of cells was 10^10^ to 10^11^ CFU/ml. One-tenth ml of each resuspended cell culture was inoculated by distributing as 10 droplets onto a pieces of stainless steel (2 × 5 cm, No. 4) with a micropipettor. The inoculated stainless steel pieces were dried inside a biosafety hood for 1 h. The final cell concentration was approximately 10^7^ to 10^8^ CFU/cm^2^.

### KrCl excilamp and LP lamp treatment

An excilamp filled with a KrCl gas mixture (KrCl excilamp) and a 254-nm germicidal lamp using low-pressure mercury (LP lamp) was used in this study^17^. Spectral irradiance of the 222-nm KrCl excilamp was identified in the previous study^17^. The wavelength gap between the output half-peak-intensity values was ca. 2.1 nm. Radiation intensities of the KrCl excilamp and LP lamp were measured using an UV fiber optic spectrometer (AvaSpec-ULS2048; Avantes, Eerbeek, Netherlands). Lamp dosages were calculated by multiplying irradiance values by the irradiation times. Inoculated stainless steel was treated at room temperature with the KrCl excilamp or LP lamp at equal dosages of 26.1, 69.6, 113.1, and 156.6 mJ/cm^2^. For microbial enumeration, each treated stainless steel piece immediately transferred into a sterile plastic tube (Corning Science Mexico, Reynosa, Mexico) containing 30 ml of sterile 0.2% peptone water and 3 g glass beads (425–600 μm; Sigma–Aldrich, St. Louis, MO, USA) and homogenized for 2 min using a vortex mixer (WISE Mix VM-10, DAIHAN Scientific Co., Seoul, Korea) at a maximum speed^38^. After homogenization, 1 ml samples were 10-fold serially diluted with 9 ml of sterile 0.2% peptone water and 0.1 ml of stomached or diluted samples were spread plated onto Sorbitol MacConkey (SMAC) agar (Difco). All plates were incubated at 37 °C for 24 h before counting colonies characteristic of the pathogens. Phenol red agar base with 1% sorbitol (SPRAB; Difco) was used to recover injured cells of *E. coli* O157:H7. After incubation at 37 °C for 24 h, typical white colonies characteristic of *E. coli* O157:H7 were enumerated. Randomly selected isolates from SPRAB plates were subjected to serological confirmation as *E. coli* O157:H7 (RIM, *E. coli* O157:H7 latex agglutination test; Remel, Lenexa, KS), because SPRAB is not typically used as a selective agar for enumerating *E. coli* O157:H7. The number of microorganisms in the untreated samples was used as control.

### Bactericidal mechanism

Lipid oxidation values were observed as the method described by Kang and Kang^31^. The quantity of malondialdehyde (MDA) was analyzed using the OxiSelect TBARS Assay Kit (Cell BioLabs, Inc., San Diego, CA), following the manufacturer’s directions. One milliliter of treated sample was centrifuged at 10,000 × g for 10 min and the supernatant was discarded and the pellet resuspended in Phosphate Buffered Saline(PBS) containing butylated hydroxytoluene (BHT). Each sample was mixed with 100 µl SDS lysis solution and incubated at room temperature for 5 min. Following incubation, each sample was reacted with 250 µl of TBA reagent at 95 °C for 1 h and cooled to room temperature in an ice bath for 5 min. Fluorescence of the sample was measured with a spectrofluorophotometer (Spectramax M2e; Molecular Devices, Sunnyvale, CA) at excitation and emission wavelengths of 540 and 590 nm, respectively. DNA damage values were observed according to the method described by Han *et al*.^39^. Following each treatment, samples were incubated with 100 µg/ml lysozyme at 37 °C for 4 h to break the cell envelope. Then, the solutions were incubated with SYBR green I (1:10,000 dilution; Sigma-Aldrich Ltd., Dublin, Ireland) at working concentration (1:1) for 15 min at 37 °C. Fluorescence of the aliquot of each sample was measured with a spectrofluorophotometer (Spectramax M2e; Molecular Devices, Sunnyvale, CA) at excitation and emission wavelengths of 485 and 525 nm, respectively.

### RNA sequencing analysis

Total RNA of *E. coli* O157:H7 ATCC 35150 grown under different conditions was extracted using the miRNeasy Mini Kit (Qiagen Inc., Hilden, Germany) according to the manufacturer’s protocol. Transcriptome analysis was performed with the pathogen grown to early stationary phase, which was 52, 12, and 7 h for 15, 25, and 37 °C, respectively. The total RNA was treated with Rnase-Free Dnase I (Qiagen Inc) for 20 min at room temperature to remove residual DNA. Namely, mRNA molecules were purified and fragmented from 2 µg of total RNA using oligo (dT) magnetic beads. The fragmented mRNA was synthesized as single-stranded cDNAs through random hexamer priming. By applying this as a template for second strand synthesis, double-stranded cDNA was prepared. After sequential process of end repair, A-tailing and adapter ligation, cDNA libraries were amplified with the Polymerase Chain Reaction (PCR). Quality of these cDNA libraries was evaluated with the Agilent 2100 BioAnlyzer (Agilent, CA, USA). They were quantified with the KAPA library quantification kit (Kapa Biosystems, MA, USA) according to the manufacturer’s library quantification protocol. Following cluster amplification of denatured templates, sequencing was progressed as paired-end (2 × 100 bp) using Illumina HiSeq2500 (Illumina, CA, USA).

### Transcriptome data analysis

Low quality reads were filtered according to the following criteria: reads containing more than 10% of skipped bases (marked as ‘N’s); reads containing more than 40% of bases whose quality scores were less than 20; and reads of which average quality scores of each read was less than 20. The whole filtering process was performed using the in-house scripts. Filtered reads were mapped to the reference genome related to the species using the aligner STAR v.2.4.0b^40^.

Gene expression level was measured with Cufflinks v2.1.1^41^ using the gene annotation database of the species. The non-coding gene region was excluded from gene expression measurement using the– mask option. To improve the accuracy of the measurement, multi-read-correction and frag-bias-correct options were applied. All other options were set to default values.

For DEG analysis, gene level count data were generated using HTSeq-count v0.6.1p1^42^ with the option “-m intersection-nonempty” and “-r option considering paired-end sequence”. Based on the calculated read count data, DEG were identified using the R package called TCC^43^. The TCC package applies robust normalization strategies to compare tag count data. Normalization factors were calculated using the iterative DEGES/edgeR method. Q-value was calculated based on the P-value using the p.adjust function of the R package with default parameter settings. The DEGs were identified based on a q value threshold less than 0.05 for correcting errors caused by multiple-testing^44^.

The GO database classifies genes according to the three categories of Biological Process (BP), Cellular Component (CC) and Molecular Function (MF) and provides information on the function of genes. To characterize the identified genes from DEG analysis, the GO based trend test was carried out through the Fisher’s exact test^45^. Selected genes of P-values < 0.001 following the test were regarded as statistically significant. For pathway analysis, genes of q-value < 0.01 and at least a two-fold change (FC ≥ 2)were required. Pathway analysis of the selected genes was performed using the KEGG database (http://www.genome.ad.jp/kegg)^46–48^, and it was permitted to publish the KEGG database. The sequence reads in this study were submitted to the NCBI SRA database under study number SRP150528 (https://www.ncbi.nlm.nih.gov/sra/SRP150528).

### Membrane lipid composition

Membrane fatty acid profiles of *E. coli* O157:H7 ATCC 35150 grown at different temperatures were analyzed. *E. coli* O157:H7 ATCC 35150 was incubated to early stationary phase and collected by centrifugation at 4,000 × g for 20 min at 4 °C. Each pellet was subjected to fatty acid extraction as described in MIDI Technical note no. 101^49^. Mixtures of hexane and methyl tert-butyl ether were used to extract the fatty acid methyl esters (FAMEs). FAMEs in the upper phase were analyzed on an Agilent gas chromatograph (model 7890A, Agilent Technologies, Santa Clara, CA, USA) equipped with a split-capillary injector and a flame ionization detector^50^. Separations were obtained using a DB-23 column (60 mm × 0.25 mm I. d., 0.25 um, Agilent Technologies). The injector temperature was set at 250 °C, the column oven at 50 °C for 1 min, followed by increasing at a rate of 15 °C/min to 130 °C, 8 °C/min to 170 °C, and 2 °C/min to 215 °C, and holding for 10 min. Hydrogen, air, and helium were used as the carrier gas, and the flow rate was set to 35 mL/min, 350 mL/min, and 35 mL/min, respectively. The detector temperature was held at 280 °C. Supelco 37 component FAME mix (Supelco, Inc., PA, USA) was used for analyzing fatty acid profiles.

### Statistical analysis

All experiments except transcriptome analysis were replicated three times. Transcriptome analysis were replicated. All data were analyzed by the analysis of variance procedure of the Statistical Analysis System (version 9.3, SAS Institute, Cary, NC) and mean values were separated using Duncan’s multiple-range test. Significant differences in the processing treatments were determined at a significance level of *p* = 0.05.

## Supplementary information

Supplementary Table

## References

[1] Cooley M (2007). Incidence and tracking of *Escherichia coli* O157: H7 in a major produce production region in California. PloS one.

[2] Tuttle J (1999). Lessons from a large outbreak of *Escherichia coli* O157: H7 infections: insights into the infectious dose and method of widespread contamination of hamburger patties. Epidemiology and Infection.

[3] Willshaw G (1994). Vero cytotoxin‐producing *Escherichia coli* O157 in beefburgers linked to an outbreak of diarrhoea, haemorrhagic colitis and haemolytic uraemic syndrome in Britain. Letters in Applied Microbiology.

[4] Bae, Y. M. & Lee, S. Y. Inhibitory effects of UV treatment and a combination of UV and dry heat against pathogens on stainless steel and polypropylene surfaces. *Journal of Food Science* **77** (2012). 10.1111/j.1750-3841.2011.02476.x 22132742

[5] Beuchat LR, Ryu J-H (1997). Produce handling and processing practices. Emerging Infectious Diseases.

[6] Wilks S, Michels H, Keevil C (2005). The survival of *Escherichia coli* O157 on a range of metal surfaces. International Journal of Food Microbiology.

[7] Maule, A. Survival of verocytotoxigenic *Escherichia coli* O157 in soil, water and on surfaces. *Journal of Applied Microbiology* **88** (2000). 10.1111/j.1365-2672.2000.tb05334.x 10880181

[8] Stokes OW (1999). Corrosion in stainless-steel and nickel-titanium files. Journal of Endodontics.

[9] Kim T, Silva J, Chen T (2002). Effects of UV irradiation on selected pathogens in peptone water and on stainless steel and chicken meat. Journal of Food Protection.

[10] Sommers CH, Sites JE, Musgrove M (2010). Ultraviolet light (254 nm) inactivation of pathogens on foods and stainless steel surfaces. Journal of Food Safety.

[11] Zimmer J, Slawson R (2002). Potential repair of *Escherichia coli* DNA following exposure to UV radiation from both medium-and low-pressure UV sources used in drinking water treatment. Applied and Environmental Microbiology.

[12] Mackey TK, Contreras JT, Liang BA (2014). The Minamata Convention on Mercury: Attempting to address the global controversy of dental amalgam use and mercury waste disposal. Science of the Total Environment.

[13] Lomaev MI (2003). Excilamps: efficient sources of spontaneous UV and VUV radiation. Physics-Uspekhi.

[14] Matafonova G, Batoev V, Astakhova S, Gomez M, Christofi N (2008). Efficiency of KrCl excilamp (222 nm) for inactivation of bacteria in suspension. Letters in Applied Microbiology.

[15] Orlowska M, Koutchma T, Kostrzynska M, Tang J (2015). Surrogate organisms for pathogenic O157: H7 and non-O157 *Escherichia coli* strains for apple juice treatments by UV-C light at three monochromatic wavelengths. Food Control.

[16] Yin F, Zhu Y, Koutchma T, Gong J (2015). Inactivation and potential reactivation of pathogenic *Escherichia coli* O157: H7 in apple juice following ultraviolet light exposure at three monochromatic wavelengths. Food Microbiology.

[17] Ha J-W, Lee J-I, Kang D-H (2017). Application of a 222-nm krypton-chlorine excilamp to control foodborne pathogens on sliced cheese surfaces and characterization of the bactericidal mechanisms. International Journal of Food Microbiology.

[18] Jaenicke R (1991). Protein stability and molecular adaptation to extreme conditons. European Journal of Biochemistry.

[19] Russell N, Fukunaga N (1990). A comparison of thermal adaptation of membrane lipids in psychrophilic and thermophilic bacteria. FEMS Microbiology Letters.

[20] Ratkowsky D, Olley J, McMeekin T, Ball A (1982). Relationship between temperature and growth rate of bacterial cultures. Journal of Bacteriology.

[21] Arsène F, Tomoyasu T, Bukau B (2000). The heat shock response of *Escherichia coli*. International Journal of Food Microbiology.

[22] Graumann P, Marahiel MA (1996). Some like it cold: response of microorganisms to cold shock. Archives of Microbiology.

[23] Ahmed NM, Conner DE, Huffman DL (1995). Heat‐Resistance of *Escherichia Coli* O157: H7 in Meat and Poultry as Affected by Product Composition. Journal of Food Science.

[24] Yuk H-G, Marshall DL (2003). Heat adaptation alters *Escherichia coli* O157: H7 membrane lipid composition and verotoxin production. Applied and Environmental Microbiology.

[25] Carruthers MD, Minion C (2009). Transcriptome analysis of *Escherichia coli* O157: H7 EDL933 during heat shock. FEMS microbiology letters.

[26] Kocharunchitt C, King T, Gobius K, Bowman JP, Ross T (2012). Integrated transcriptomic and proteomic analysis of the physiological response of *Escherichia coli* O157: H7 Sakai to steady-state conditions of cold and water activity stress. Molecular & Cellular Proteomics.

[27] Wang S (2009). Transcriptomic response of *Escherichia coli* O157: H7 to oxidative stress. Applied and Environmental Microbiology.

[28] Jenkins D, Schultz J, Matin A (1988). Starvation-induced cross protection against heat or H_2_O_2_ challenge in *Escherichia coli*. Journal of Bacteriology.

[29] Rees CE, Dodd CE, Gibson PT, Booth IR, Stewart GS (1995). The significance of bacteria in stationary phase to food microbiology. International Journal of Food Microbiology.

[30] Clauss M, Grotjohann N (2008). Effective Photoinactivation of Alpha‐Amylase, Catalase and Urease at 222 nm Emitted by an KrCl‐Excimer Lamp. CLEAN–Soil, Air, Water.

[31] Kang J-W, Kim S-S, Kang D-H (2018). Inactivation dynamics of 222 nm krypton-chlorine excilamp irradiation on Gram-positive and Gram-negative foodborne pathogenic bacteria. Food Research International.

[32] Gomez M (2010). Testing a KrCl excilamp as new enhanced UV source for 4-chlorophenol degradation: Experimental results and kinetic model. Chemical Engineering and Processing: Process Intensification.

[33] Stancik LM (2002). pH-dependent expression of periplasmic proteins and amino acid catabolism in *Escherichia coli*. Journal of Bacteriology.

[34] Schaffer JE, Lodish HF (1994). Expression cloning and characterization of a novel adipocyte long chain fatty acid transport protein. Cell.

[35] Farewell A, Diez AA, DiRusso CC, Nyström T (1996). Role of the *Escherichia coli FadR* regulator in stasis survival and growth phase-dependent expression of the *uspA*, *fad*, and *fab* genes. Journal of Bacteriology.

[36] Shaw MK, Ingraham JL (1965). Fatty acid composition of *Escherichia coli* as a possible controlling factor of the minimal growth temperature. Journal of Bacteriology.

[37] Russell NJ (2002). Bacterial membranes: the effects of chill storage and food processing. An overview. International Journal of Food Microbiology.

[38] Ban G-H, Kang D-H (2016). Effect of sanitizer combined with steam heating on the inactivation of foodborne pathogens in a biofilm on stainless steel. Food Microbiology.

[39] Han L (2016). Mechanisms of inactivation by high-voltage atmospheric cold plasma differ for *Escherichia coli* and *Staphylococcus aureus*. Applied and Environmental Microbiology.

[40] Dobin A (2013). STAR: ultrafast universal RNA-seq aligner. Bioinformatics.

[41] Trapnell C (2010). Transcript assembly and quantification by RNA-Seq reveals unannotated transcripts and isoform switching during cell differentiation. Nature Biotechnology.

[42] Anders S, Pyl PT, Huber W (2014). HTSeq–a Python framework to work with high-throughput sequencing data. Bioinformatics.

[43] Sun J, Nishiyama T, Shimizu K, Kadota K (2013). TCC: an R package for comparing tag count data with robust normalization strategies. BMC bioinformatics.

[44] Benjamini, Y. & Hochberg, Y. Controlling the false discovery rate: a practical and powerful approach to multiple testing. *Journal of the Royal Statistical Society*. *Series B (Methodological)*, 289–300 (1995).

[45] Fisher RA (1922). On the interpretation of χ 2 from contingency tables, and the calculation of P. Journal of the Royal Statistical Society.

[46] Kanehisa M, Goto S (2000). KEGG: kyoto encyclopedia of genes and genomes. Nucleic acids research.

[47] Kanehisa M, Sato Y, Kawashima M, Furumichi M, Tanabe M (2015). KEGG as a reference resource for gene and protein annotation. Nucleic acids research.

[48] Kanehisa M, Furumichi M, Tanabe M, Sato Y, Morishima K (2016). KEGG: new perspectives on genomes, pathways, diseases and drugs. Nucleic acids research.

[49] Sasser, M. Identification of bacteria by gas chromatography of cellular fatty acids (1990).

[50] Garces R, Mancha M (1993). One-step lipid extraction and fatty acid methyl esters preparation from fresh plant tissues. Analytical Biochemistry.

